# Supplementation with Small-Quantity Lipid-Based Nutrient Supplements Does Not Increase Child Morbidity in a Semiurban Setting in Ghana: A Secondary Outcome Noninferiority Analysis of the International Lipid-Based Nutrient Supplements (iLiNS)-DYAD Randomized Controlled Trial

**DOI:** 10.1093/jn/nxz243

**Published:** 2019-09-11

**Authors:** Seth Adu-Afarwuah, Rebecca R Young, Anna Lartey, Harriet Okronipa, Per Ashorn, Ulla Ashorn, Brietta M Oaks, Kathryn G Dewey

**Affiliations:** 1 department of Nutrition and Food Science, University of Ghana, Legon, Accra, Ghana;; 2 Program in International and Community Nutrition, Department of Nutrition, University of California, Davis, CA, USA;; 3 Centre for Child Health Research, Tampere University Faculty of Medicine and Health Sciences and Tampere University Hospital, Tampere, Finland; and; 4 Department of Nutrition and Food Sciences, University of Rhode Island, Kingston, RI, USA

**Keywords:** maternal-infant supplementation, multiple micronutrient supplements, lipid-based nutrient supplements, infant morbidity, child morbidity

## Abstract

**Background:**

Adequate knowledge about the safety of consumption of small-quantity lipid-based nutrient supplements (SQ-LNSs) is needed.

**Objective:**

We aimed to test the hypothesis that SQ-LNS consumption is noninferior to control with respect to child morbidity

**Methods:**

Women (*n* = 1320) ≤20 wk pregnant were assigned to iron and folic acid until delivery with no supplementation for offspring; or multiple micronutrient supplements until 6 mo postpartum with no supplementation for offspring; or SQ-LNSs until 6 mo postpartum, and SQ-LNSs for offspring (6 mg Fe/d) from 6 to 18 mo of age [the lipid-based nutrient supplement (LNS) group]. We assessed noninferiority (margin ≤20%) between any 2 groups during 0-6 mo of age, and between the non-LNS and LNS groups during 6-18 mo of age for caregiver-reported acute respiratory infection, diarrhea, gastroenteritis, fever/suspected malaria, poor appetite, and “other illnesses.”

**Results:**

During 0-6 mo of age, 1197 infants contributed 190,503 infant-days. For all morbidity combined, overall mean incidence (per 100 infant-days) was3.3 episodes, overall mean prevalence (percentage of infant-days) was 19.3%, and the 95% CIs of the incidence rate ratio (IRR) and longitudinal prevalence rate ratio (LPRR) between any 2 groups were ≤1.20. During 6-18 mo, there were 240,097 infant-days for the non-LNS group and 118,698 for the LNS group. For all morbidity combined, group mean incidences were 4.3 and 4.3, respectively (IRR: 1.0; 95% CI: 1.0, 1.1), and mean prevalences were 28.2% and 29.3%, respectively (LPRR: 1.0; 95% CI: 1.0,1.1). Noninferiority was inconclusive for diarrhea, fever/suspected malaria, and poor appetite.

**Conclusions:**

SQ-LNS consumption does not increase reported overall child morbidity in this population compared with the 2 other treatments. This trial was registered at clinicaltrials.gov as NCT00970866. *J Nutr* 2019;00:1-12.

## Introduction

In low-income settings, the diets of many people are based predominantly on cereals and tubers and are of low nutrient quality (1). The consumption of low-quality diets is associated with the delivery of low-birth-weight and small-for-gestational- age (SGA) infants among pregnant women (2-4) and with poor growth and development among children (5). Currently, efforts to increase the nutrient intakes of women and children in low- income settings are an international priority (6, 7).

The International Lipid-based Nutrient Supplements (iLiNS) Project developed small-quantity lipid-based nutrient supplements (SQ-LNSs) for enriching home-prepared foods for women and infants in low-income settings (8), as a potential strategy to increase nutrient intakes during the “first 1000 days.” A Cochrane review suggests that in women, prenatal SQ- LNS supplementation, compared with iron and folic acid (IFA), has positive effects on birth outcomes, including greater infant weight and length at birth, and lower prevalence of SGA and newborn stunting (9). Another Cochrane review shows that in children 6-23 mo of age, the consumption of SQ-LNSs added to complementary foods improves growth and anemia status when compared with no intervention, and may be more effective than other alternatives such as fortified blended foods and multiple micronutrient powders at improving growth (10).

Despite the positive meta-analysis evidence so far reported (10), a possible concern about SQ-LNS supplementation for children is that it might be associated with increased risk of morbidity, including malaria, as a result of the supplemental iron provided (11). Adequate knowledge about the safety of SQ-LNS supplementation among infants and young children is needed to inform future efforts to scale up the use of such products. So far, the morbidity results from previous child lipid-based nutrient supplement (LNS) supplementation trials have been mixed. In several studies, no differences between LNS and control groups were reported for diarrhea (12-14), runny nose (12), fever (12), respiratory infections (12, 14, 15), and malaria (13, 15). Elsewhere, LNS supplementation reduced diarrhea (16, 17), fever (16, 18), and runny nose (18), whereas in other settings, it increased diarrhea (18) and possibly malaria-related nonscheduled visits to a health center (19). Furthermore, we are aware of only 1 report on child morbidity thus far (20) from a trial that included a group exposed to LNS both prenatally (maternal supplementation) and postnatally (maternal and infant supplementation).

The iLiNS-DYAD trial in Ghana included both pre- and postnatal SQ-LNS consumption. In Ghana, child undernutrition has decreased in recent years (21), but micronutrient deficiencies (22) and childhood morbidity such as diarrhea and respiratory infections (23) remain common. We reported previously that SQ-LNS consumption increased fetal growth (24) as well as child growth (25) and iron status (26) by 18 mo of age. Herein, we performed a secondary outcome, noninferiority analysis to determine whether SQ-LNS supplementation was noninferior to the 2 other supplementation regimens with respect to child morbidity. We hypothesized that the incidence or longitudinal prevalence of childhood morbidity among infants provided with SQ-LNSs from 6 to 18 mo of age, and whose mothers were provided with SQ-LNSs during pregnancy and the first 6 mo postpartum, would not be higher than those among infants who received no supplements from 6 to 18 mo of age, and whose mothers received IFA during pregnancy only, or multiple micronutrient supplements (MMNs) during pregnancy and lactation.

## Methods

### Trial setting, design, and ethical approval

We have described the iLiNS-DYAD Ghana trial (NCT00970866) in previous articles (24, 25). With the aim of evaluating the efficacy of SQ-LNSs for pregnant and lactating women and their infants, the trial was conducted in the semiurban setting stretching from Somanya to Kpong in the Eastern Region ~70 km north of Accra, and was designed as a partially double-blind, individually randomized, controlled trial with 3 equal-size groups. Women attending antenatal clinics in 4 main health centers in the area were eligible if they were ≥18 y old and ≤20 wk pregnant at the time of enrollment. Exclusion criteria were being a nonresident of the area; intention to relocate from the area before the expected end of the trial; milk or peanut allergy; participation in another trial; HIV infection; asthma; epilepsy; tuberculosis; any malignancy; or unwillingness to participate in trial procedures. Women signed or thumb-printed informed consents for their own participation, and for their infants if born alive.

At enrollment, women were randomly assigned to the 3 groups as follows: *1*) the IFA group, assigned to consume 60 mg Fe/d + 400 *µ*g folic acid/d supplements during pregnancy and 200 mg Ca/d as placebo during the first 6 mo postpartum, and no supplementation for offspring; 2) the MMN group, assigned to consume MMNs until 6 mo postpartum, and no supplementation for offspring; and 3) the LNS group, assigned to consume SQ-LNSs until 6 mo postpartum, and supplementation for the offspring with SQ-LNSs designed for infants from 6 to 18 mo of age. All supplements were intended for daily consumption.

The trial was approved by 3 ethics committees (University of California, Davis; Ghana Health Service; and Noguchi Memorial Institute for Medical Research) and monitored by a Data and Safety Monitoring Board.

### Trial supplements

As reported previously (24, 25), the IFA reflected the Ghana Health Service’s standard micronutrient supplementation for pregnant women at the time of the study (27). The MMN consisted of 18 vitamins and minerals at 1 or 2 times the RDA for pregnancy, except for iron, for which we used 20 mg/d instead of the 30 mg/d in the UNIMMAP formulation (28)or the 30-60 mg/d in the WHO guideline (27), because of previous evidence (29) showing that 20 mg Fe/d was as effective as 40 mg Fe/d or 80 mg Fe/d in treating anemia during pregnancy but less likely to cause gastrointestinal side effects than a 30-60 mg/d dose. The SQ-LNS designed for pregnant and lactating women (SQ-LNS-P&L) had the same micronutrient contents as the MMN, and in addition, energy, protein, essential fatty acids, and the maximum amounts of calcium, magnesium, phosphorus, and potassium that could be included given technical and organoleptic constraints (8). With the 20 mg Fe/d in the SQ-LNS-P&L, it was possible to have just 1 product for both pregnancy and lactation, assuming that this dose, in addition to iron from the usual diet, would give a total daily intake that was close to the amount in the UNIMMAP formulation (28) and would therefore meet the RDA of 27 mg Fe for pregnancy, while at the same time, it would not greatly exceed the RDA (9 mg/d) for lactation (8, 30).

The daily dose of the SQ-LNS designed for infants (SQ-LNS-INF) contained the WHO, WHO/FAO, and FAO Recommended Nutrient Intakes (RNIs) for key micronutrients for infants 7-12 mo of age, with a few exceptions (8): the 6.0 mg Fe/d was slightly lower than the WHO/FAO RNI (6.2 mg/d) and 33% lower than the dose previously used in Ghana (12), because of concerns about increasing the risk of malaria and infections in an area of high malaria endemicity (31); and the 8 mg Zn/d content (8) was higher than the WHO/FAO RNI (2.5 mg/d) because of our previous study (12) showing that zinc absorption may be decreased in the predominantly maize-based diet high in phytate typically consumed by infants during the complementary feeding period.

The IFA and MMN supplements as well as the calcium placebo were provided as capsules in blister packs of 10, whereas the SQ-LNS-P&L was provided in 20-g sachets, and the SQ-LNS-INF was provided in 10-g sachets. All supplements were meant for daily consumption.

### Initial screening, baseline assessments, group assignments, and blinding

We determined women’s eligibility by using a screening questionnaire, and with the aid of information recorded from their antenatal cards. For eligible women agreeing to participate, we collected baseline information, including sociodemographic characteristics; gestational age (by using ultrasound biometry, Aloka SSD 500); anthropometric status (by using standard procedures); blood hemoglobin (Hb) concentration (HemoCue AG); zinc protoporphyrin concentration (Hematofluorometer, Aviv Biomedical Co.), and peripheral malaria parasitemia (Vision Biotech) (24).

Women were randomly assigned after the baseline assessments, as follows (24, 25): *1*) the study statistician at University of California, Davis, Janet M Peerson, developed the group assignments in blocks of 9 (SAS for Windows version 9.4); *2*) the study data manager at the University of Ghana, who was not involved in enrollment, prepared opaque envelopes containing the assignments, which were numbered and stacked by block number; and *3*) the study nurse at the field site performed the random assignment. At each enrollment, the study nurse shuffled 9 envelopes taken from the top of the stack and asked the participant to make a pick to reveal the group assignment. The nurse then returned the unused envelopes to the top of the stack. When there were <9 women left to be enrolled, the nurse shuffled whatever number of envelopes remained. Any allocation information was kept securely by the field supervisor in Ghana and the study statistician at University of California, Davis, only.

At enrollment, the study nurse gave women a 2-wk supply of supplements, advice to take 1 capsule/d with water after a meal or one 20-g SQ-LNS-P&L sachet/d mixed with food, and a standard nutrition message: “Do not forget to eat meat, fish, eggs, fruits, and vegetables whenever you can; you still need these foods even as you take the supplements we have given you” (24, 25). We color-coded all blister packs, so that the study team and participants knew the IFA and MMN capsules only by their color codes (3 colors for the IFA group and 3 for the MMN group). We could not blind study workers and participants with regard to which women received SQ-LNSs and which received the capsules, but laboratory staff who collected any samples or performed any laboratory analyses had no knowledge of the group assignments, and the randomization code was broken only after the analyses of the primary outcomes had been completed.

### Follow-up procedures

Field workers visited pregnant women in their homes biweekly to provide a fresh supply of supplements and monitor supplement intakes. All live-born singleton infants were enrolled into the study. In twin births, 1 infant was randomly selected during the first visit immediately after birth, and the selected infant was enrolled if he or she was born alive. After giving birth, women and their infants were visited weekly, but the women received their supplements or placebo and had intakes of these monitored biweekly as before.

Women exited the study at 6 mo postpartum, but the weekly visits for the infants continued. At 6 mo of age, the study nurse gave the “minimum message” on complementary feeding to all mothers at the laboratory after the infants had participated in their first laboratory assessment: “Breastfeed your baby as you did before 6 mo of age; do not forget to feed your baby meat, fish, eggs, fruits, and vegetables whenever you can.” During the next usual weekly home visit, field workers delivered the first supply of SQ-LNS-INFs for infants in the LNS group, advised the mothers or caregivers on how to feed the supplements (i.e., mix the entire content of 1 sachet with 2-3 tablespoons of food for the infant before feeding additional foods if the infants desire, 2 times each day), and repeated the “minimum message” on complementary feeding given by the study nurse. For infants in the other 2 groups not assigned to any supplements, field workers only repeated the “minimum message” to the mothers or caregivers. Field workers delivered a fresh supply of infants’ SQ-LNSs during the usual weekly visits, but the “minimum message” was not repeated again thereafter. Infants exited the study at 18 mo of age.

### Measurement of outcome variables

At each weekly home visit for study infants, field workers collected caregivers’ reports of common morbidity symptoms experienced by the infants, as well as the infants’ nonscheduled visits to any “treatment points” with the purpose to receive treatment of illness, during the period since the last visit or the last 10 d, if the current visit was delayed for >10 d. We defined “treatment points” to include hospitals, clinics, health posts, private physicians, pharmacists, nurses, midwives, drug stores not operated by pharmacists, and traditional healers, because residents of the study area typically seek treatment of illnesses from all such quarters, and therefore focusing on hospitals and clinics alone would underestimate treatments of morbidity. Whenever an infant was reportedly taken for a nonscheduled visit to a treatment point because of illness, the field worker asked about the specific illnesses that precipitated the nonscheduled visit.

To facilitate data collection, field workers trained caregivers to use a calendar grid with pictures in its rows depicting the common morbidity symptoms (diarrhea, vomiting due to illness, fever, cough, nasal discharge, and poor appetite) and a visit to a treatment point, to record information. The calendar grid had columns for recording information for each day ≤10 d. At each visit, field workers transferred the information recorded by the caregiver on the calendar grid onto a morbidity form after verification with the caregiver, and in addition, asked if the child had rapid breathing or difficult breathing on any day during the period. As much as possible, all morbidity data were collected using the daily calendar grid. As a complementary strategy, we asked fathers, older children, and other key members within the households to remind mothers about entering the children’s morbidity information on the calendar each day. With the intense weekly home visits by field workers, the filling out of the morbidity calendar soon became the normal daily routine for mothers and families. If a mother missed filling out the calendar grid, it was generally for a few days within the week and not the entire week or any longer period of time. If, during the weekly home visit, a field worker found that a mother had missed filling out the calendar on some days, the field worker probed the mother to determine if the child had any morbidity symptoms or was taken to any treatment centers on those days.

Several trials across Africa have used a calendar grid to collect children’s morbidity data, including some in Malawi (15, 19) and South Africa (18), as well as our own earlier trial in Ghana (12). Work by Arnold et al. (32) testing the use of the daily calendar in 5 countries to determine the optimal recall period for caregiver-reported illness in children < 24 mo of age recommended the use of a 7-d (weekly) recall period compared with a 2-d recall period, owing to the increased statistical efficiency (e.g., reduced sample sizes required) that resulted from the use of the former.

The morbidity outcome variables were evaluated for 2 time periods, namely *1*) 0-6 mo of age [the period when the WHO recommends exclusive breastfeeding (33), during which no infants directly consumed any study supplements] and 2) 6-18 mo of age (the period of complementary feeding during which infants in the LNS group consumed SQ-LNSs, and those in the IFA and MMN groups did not consume any study supplements). The outcome variables were *1* ) the incidence and longitudinal prevalence of caregiver-reported common morbidity episodes, and *2*) the incidence of caregiver-reported nonscheduled visits to a “treatment point” because of illness. The morbidity episodes were *1* ) acute respiratory infection (ARI), *2*) diarrhea (caregiver-defined), *3*) gastroenteritis, *4*) fever or suspected malaria, *5*) “other illnesses,” and *6*) poor appetite. We separated caregivers’ diagnosis of diarrhea from gastroenteritis or the clinical definition of diarrhea (34) because of evidence that there may be a discrepancy between caregivers’ diagnosis of diarrhea and the one defined by the clinical criteria (35). The diagnoses of gastroenteritis, ARI, and suspected malaria were derived from a combination of symptoms occurring for ≥1 d, while ensuring that diagnoses were mutually exclusive. We defined acute gastroenteritis as vomiting or diarrhea for ≥3d, or both for ≥1 d (36); diarrhea as passage of ≥3 loose stools in the past 24 h (34) regardless of other symptoms; ARI as cough, rapid breathing, difficult breathing, or nasal discharge in the absence of diarrhea regardless of fever; fever or suspected malaria as increased body temperature in the absence of diarrhea and ARI, with or without other symptoms; and “other illnesses” as other symptoms in the absence of diarrhea, ARI, or fever. For all illnesses, we defined an episode as the period starting from the day the child had symptoms when preceded by ≥2 d of no symptoms or data. An episode lasted until the last day the child had symptoms which was then followed by ≥2 symptom-free days (19). The number of days that a child was at risk of each of the symptoms (i.e., not already exhibiting that symptom) was calculated, accounting for the mutually exclusive definitions.

We calculated the incidence of a morbidity episode as the number of episodes divided by the total days at risk, and longitudinal prevalence as the percentage of follow-up days that the infant had the illness (37). The incidence of nonscheduled visits to a treatment point was defined as the total number of nonscheduled visits divided by the total number of follow-up days. We calculated the incidence of treatment point visits due to all morbidity events combined, as well as the incidence of treatment point visits due to the specific morbidity events that precipitated the visits. We used “other reasons” for treatment point visits to represent visits made that were not due to any of the morbidity events listed.

### Sample size and data analysis

The sample size for the iLiNS-DYAD-Ghana trial (24, 25) was based on detecting an effect size or Cohen’s *d* (38) of ≥0.3 between any 2 groups for the primary continuous outcomes (child length at birth and child length by 18 mo of age), with a 2-sided 5% test and 80% power, given 3 intervention groups. A total of 1320 women were enrolled after a temporary mislabeling of some IFA and MMN supplements, which we reported previously (24, 25).In our trial protocol at clinicaltrials.org, as well as the general statistical analysis plan (SAP) for the iLiNS-DYAD- Ghana trial, which we posted at our website (www.ilins.org) before any analyses began, we listed child morbidity as one of the secondary outcomes to be analyzed separately. An additional, more detailed SAP was developed specifically for this present analysis and finalized before data analysis began. We performed all analyses by using SAS version 9.4 (SAS Institute) and the complete case intention-to-treat principle (39). That is, data for all infants were included in the analysis, regardless of the number of days supplements were consumed by the infants or their mothers. Children who dropped out of the study were included in the analysis up until the date of dropout or last known date of morbidity information.

As reported previously, the self-reported adherence to supplement intake for women (pregnancy/lactation) was 88.1%/85.7% for the IFA group, 87.0%/85.0% for the MMN group, and 83.7%/80.0% for the LNS group (40), and that for the infants in the LNS group was 73.5% (25).

We summarized background maternal and household variables by intervention groups according to the supplements women were assigned to at enrollment, as done previously (24, 25). For each outcome variable, we calculated the mean incidence or longitudinal prevalence by group, and compared group mean incidence or longitudinal prevalence by using noninferiority testing (15, 19). For comparing mean incidence, we calculated the incidence rate ratio (IRR—ratio of the incidence rate in the treatment group to the incidence rate in the comparison group), along with its 95% CI. Incidence and IRR (95% CI) were generated using negative binomial regression models (SAS PROC GLIMMIX). The negative binomial models used an offset to adjust for the number of follow-up days for each child. For comparing mean longitudinal prevalence, we calculated longitudinal prevalence rate ratio (LPRR) as the ratio of the longitudinal prevalence in the intervention group to the longitudinal prevalence in the control group, and its 95% CI. Longitudinal prevalence and LPRR (95% CI) were generated using log- linear least-squares regression (SAS PROC GLIMMIX).

For the noninferiority testing, we predefined a noninferiority margin (δ) of no more than a 20% increase in an outcome for the intervention group compared with the control group. There is no standard noninferiority margin for assessing safety in micronutrient supplementation trials for children, but a similar margin has been used in several such contexts, including 2 trials in Bangladesh (14, 41) and 2 in Malawi (15, 19). This would mean that if a child in the non- LNS group were expected to have 5 morbidity episodes during the period from birth to 6 mo of age, or from 6 to 18 mo of age, we would tolerate only a mean of 1 additional episode during the period for children in the LNS group. The 20% margin is consistent with the 10-20% range of margins often thought of as acceptable (42), and in this case choosing the upper end of this range is consistent with various guidance documents (43, 44) and other studies (41) that support the selection of a wider noninferiority margin if a product offers an advantage in some other aspect of its profile. Based on available evidence (10), we considered that the LNSs met this condition. For any outcome, our noninferiority hypothesis was based on the comparison of the upper bound of the 2-sided 95% CI for the IRR or LPRR to the prespecified noninferiority margin of ≤1.2, in accordance with CONSORT 2010 guidelines (45).We anticipated the following possible conclusions: superiority of the intervention group compared with the control group if the upper bound of the 2-sided 95% CI for the IRR or LPRR was < 1.0; noninferiority if the upper bound of the 95% CI of the IRR or LPRR was ≤1.2; inconclusive noninferiority if the lower bound of the 95% CI of the IRR or LPRR was <1.2 and the upper bound was >1.2; or inferiority, if the lower bound of the 95% CI for the IRR or LPRR was >1.2 (45, 46).

In the analysis of outcomes during 0-6 mo of age, we compared infants in all 3 groups according to the supplements women were assigned to at enrollment, and for the analysis of outcomes during 618 mo of age, we compared infants in the LNS group (who consumed the SQ-LNSs) with those in the IFA and MMN groups combined (who did not consume any supplements). In the text, we have presented statistics on background characteristics as means ± SDs (continuous outcomes) or percentages (binary outcomes), and on the outcome variables as ratios and their 95% CIs.

## Results

The iLiNS-DYAD-Ghana trial data were collected between December, 2009 and March, 2014. At enrollment, the women’s mean age was ~27 y, they had nearly 8 y of formal education, and were ~16 wk pregnant (Table 1). Further, about one- third of the women were nulliparous, 10% tested positive in the rapid diagnostic test for malaria, and 14% were anemic when using the Hb concentration cutoff of <100 g/L (47). These background characteristics were comparable across the 3 groups.

**Table 1:** Characteristics of women (*n* = 1320) who participated in the iLiNS-DYAD-Ghana micronutrient supplementation trial, by group according to supplements women were assigned to when enrolled^1^

Characteristics at enrollment	IFA (n = 441)4	MMN (*n* = 439)	LNS (n = 440)
Age, y	26.5 ± 5.2 (441)	26.7 ± 5.7 (439)	26.9 ± 5.6 (440)
Formal education, y	7.8 ± 3.6 (441)	7.6 ± 3.5 (439)	7.6 ± 3.9 (440)
Weeks of gestation	16.2 ± 3.3 (438)	16.0 ± 3.2 (438)	16.1 ± 3.3 (435)
Asset index^2^	0.05 ± 1.01 (433)	0.05 ± 0.99 (431)	- 0.09 ± 1.00 (432)
Housing index^2^ 0.05 ± 0.98 (433)	- 0.03 ± 1.02 (431)	- 0.01 ± 1.00 (432)
FIAS score^3^	2.8 ± 4.6 (436)	2.4 ± 4.1 (429)	2.6 ± 4.0 (432)
Married or cohabiting	406/441 (92.1)	413/439 (94.1)	405/440 (92.0)
Primiparous women	162/441 (36.7)	137/439 (31.2)	147/440 (33.4)
Positive malarial RDT^4^	40/441 (9.1)	39/438 (8.9)	54/440 (12.3)
Anemic^5^	55/441 (12.5)	70/438 (16.0)	60/440 (13.6)

1 Values are mean ± SD (n) or n/total *n* (%). IFA group randomly assigned to receive 60 mg Fe and 400 *ixg* folic acid/d during pregnancy and 200 mg Ca/d as placebo during the first 6 mo postpartum; LNS group randomly assigned to receive 20 g/d small- quantity LNS until 6 mo postpartum; MMN group randomly assigned to receive 18 vitamins and minerals (including 20 mg Fe)/d until 6 mo postpartum. The small-quantity LNS had the same micronutrients as the MMN group, plus 4 more minerals (Ca, P K, and Mg) and macronutrients. HFIAS, Household Food Insecurity Access Scale; IFA, iron and folic acid; LNS, lipid-based nutrient supplement; MMN, multiple micronutrient supplement; n, number of participants whose response was “yes” for the variable in question; RDT rapid diagnostic test; total *n*, the number of participants in the group in question.

2 Proxy indicators for household socioeconomic status; higher values represent higher socioeconomic status.

3 HFIAS is a proxy indicator for household food insecurity (1); higher values represent higher food insecurity.

4 Clearview Malarial Combo, Vision Biotech.

5 Anemia defined as blood hemoglobin concentration <100 g/L (2).

**Figure 1:**
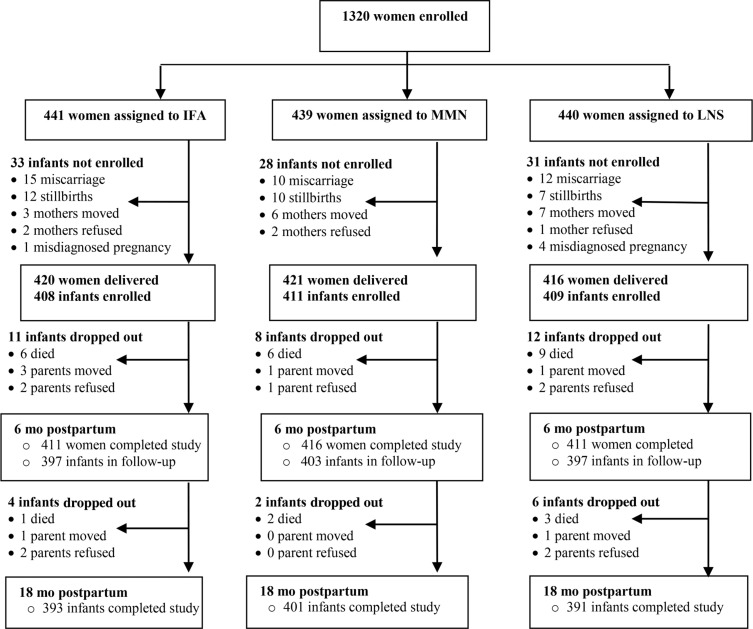
Study profile showing infants whose mothers were enrolled into the trial, and the reasons some infants were lost to follow-up. IFA group: infants were assigned to receive no supplements, whereas their mothers were assigned to receive 60 mg Fe and 400 *fig* folic acid/d during pregnancy and 200 mg Ca/d as placebo during 6 mo postpartum; MMN group: infants were assigned to receive no supplements, whereas their mothers were assigned to receive 1 multiple micronutrient capsule/d containing 18 vitamins and minerals (including 20 mg Fe/d during pregnancy and the first 6 mo postpartum); LNS group: infants were assigned to receive 20 g LNS/d (designed for infants) containing 6 mg Fe/d from 6 to 18 mo of age, whereas their mothers received 20 g LNS/d (designed for women) with the same micronutrients as the MMN group during pregnancy and the first 6 mo postpartum. Both LNS products contained 4 additional minerals (Ca, P K, and Mg) as well as macronutrients. IFA, iron and folic acid; LNS, lipid-based nutrient supplement; MMN, multiple micronutrient supplement.

A total of 1257 infants were born to the women in the study, of whom 1228 were enrolled; the remaining 29 not enrolled were stillborn (Figure 1). Nearly (*n* = 1185) all of the infants enrolled completed the study at 18 mo of age, except 27 who died, 9 whose parents refused, and 7 who relocated. Apart from having a greater mean ± SD housing index (0.3 ± 1.0 compared with -0.01 ± 1.00; *P* = 0.034), the dropouts did not differ in background characteristics from those who completed the study.

During the period 0-6 mo of age, there were 1197 infants in the study, who contributed >190,000 infant-days of follow- up for the 3 groups. During this period, the overall mean incidence and mean longitudinal prevalence of all morbidity events combined were 3.3 episodes per 100 infant-days and 19.3% of infant-days, respectively. In each of the pairwise comparisons, the 95% CIs of the IRR (Table 2) or LPRR (Table 3) for all reported morbidity events combined were ≤1.2, suggesting noninferiority between any 2 groups. Concerning specific reported morbidity outcomes, there was a similar pattern of noninferiority between groups in the IRR (Table 2) or LPRR (Table 3) for ARI. However, the upper bounds of the 95% CIs of the IRRs or LPRRs for diarrhea (caregiver-defined), gastroenteritis, fever/suspected malaria, “other illness,” and poor appetite generally exceeded the noninferiority margin of 1.20, which suggested inconclusive noninferiority between the groups in pairwise comparisons. During 0-6 mo of age, the overall mean incidence of all reported nonscheduled treatment point visits combined was 1.0 per 100 infant-days (Table 4). Most of these unscheduled visits were due to gastroenteritis, ARI, and fever/suspected malaria. In general, there was noninferiority between groups in the IRR of all nonscheduled visits combined, or nonscheduled visits due to ARI. However, there was generally inconclusive noninferiority between groups in the IRRs of nonscheduled visits due to gastroenteritis, fever/suspected malaria, “other illness,” and poor appetite.

**Table 2:** Incidence of caregiver-reported morbidity events during 0-6 mo of age among infants born to pregnant women enrolled in the iLiNS-DYAD-Ghana micronutrient supplementation trial, by group according to supplements women were assigned to when enrolled^1^

	Incidence (per 100 infant-days), by group^2^			IRR of pairwise groups (95% CI)^2^		
	IFA (63,119 infant-days)	MMN (65,054 infant-days)	LNS (62,330 infant-days)	MMN vs IFA LNS vs IFA	LNS vs MMN
All morbidity events combined	3.35	3.21	3.27	0.95 (0.85,1.07)^3^	0.98 (0.87,1.09)^3^	1.02 (0.91,1.14)^3^
Acute respiratory infections	2.53	2.40	2.40	0.95 (0.83,1.10)^3^	0.95 (0.83,1.09)^3^	1.00 (0.87,1.15)^3^
Diarrhea (caregiver-defined)	0.52	0.53	0.55	1.04 (0.81,1.34)	1.08 (0.84,1.39)	1.04 (0.81,1.33)
Gastroenteritis	0.70	0.70	0.70	1.02 (0.80,1.29)	1.02 (0.80,1.20)^3^	1.00 (0.79,1.28)
Fever/suspected malaria	0.68	0.63	0.69	0.92 (0.71,1.19)^3^	1.01 (0.78,1.29)	1.09 (0.85,1.41)
Other illness	0.10	0.10	0.11	0.97 (0.61,1.54)	1.08 (0.69, 1.68)	1.11 (0.71,1.74)
Poor appetite	0.23	0.23	0.29	1.01 (0.72,1.42)	1.29 (0.94,1.78)	1.28 (0.93,1.76)

1 *n* = 397, 403, and 397 for the IFA, MMN, and LNS groups, respectively. IFA group: infants of women assigned to 60 mg Fe/d and 400 *ixg* folic acid/d during pregnancy and 200 mg Ca/d as placebo during the first 6 mo postpartum, with no supplementation for the offspring; MMN group: infants of women assigned to multiple micronutrient capsules containing 18 vitamins and minerals (including 20 mg Fe/d) during pregnancy and the first 6 mo postpartum, with no supplementation for the offspring; LNS group: infants of women assigned to 20 g LNS/d with the same micronutrients as the MMN group during pregnancy and the first 6 mo postpartum, and with the offspring assigned to 20 g LNS/d (for infants) containing 6 mg Fe/d from 6 to 18 mo of age. The LNS products contained calcium, phosphorous, potassium, and magnesium as well as macronutrients. All supplements were intended for daily consumption. IFA, iron and folic acid; IRR, incidence rate ratio; LNS, lipid-based nutrient supplement; MMN, multiple micronutrient supplement.

2 Incidence (per 100 infant-days) was calculated by dividing the number of morbidity episodes by the total number of days at risk and multiplying the results by 100. IRR is the ratio of the incidence in the treatment group to the incidence in the comparison group. Incidence and IRR (95% CI) were generated using negative binomial regression models (SAS PROC GLIMMIX) and adjusting for the number of follow-up days for each child.

3 Noninferiority is concluded.

**Table 3:** Longitudinal prevalence of caregiver-reported morbidity events during 0-6 mo of age among infants born to pregnant women enrolled in the iLiNS-DYAD-Ghana micronutrient supplementation trial, by group according to supplements women were assigned to when enrolled^1^

	Longitudinal prevalence, by group^2^	LPRR of pairwise groups (95% CI)^2^
	IFA (63,119 infant-days)	MMN (65,054 infant-days)	LNS (62,330 infant-days)	MMN vs IFA	LNS vs IFA	LNS vs MMN
All morbidity events combined	19.60	19.08	19.40	0.95 (0.82,1.11)^3^	0.98 (0.85,1.15)^3^	1.03 (0.89,1.20)^3^
Acute respiratory infections	14.74	14.42	14.19	0.98 (0.82,1.16)^3^	0.96 (0.81,1.14)^3^	0.98 (0.83,1.17)^3^
Diarrhea (caregiver-defined)	2.67	2.86	2.90	1.07 (0.74,1.56)	1.09 (0.75,1.58)	1.01 (0.70,1.47)
Gastroenteritis	2.44	2.70	2.91	1.11 (0.79,1.54)	1.19 (0.86,1.66)	1.08 (0.78,1.50)
Fever/suspected malaria	1.35	1.12	1.32	0.84 (0.61,1.14)^3^	0.98 (0.72,1.34)	1.18 (0.86,1.60)
Other illness	0.42	0.38	0.50	0.89 (0.37, 2.14)	1.17 (0.50, 2.77)	1.31 (0.55,3.11)
Poor appetite	0.93	0.83	1.22	0.90 (0.56,1.44)	1.32 (0.82, 2.11)	1.47 (0.92, 2.35)

1 *n* = 397, 403, and 397 for the IFA, MMN, and LNS groups, respectively. IFA group: infants of women assigned to 60 mg Fe/d and 400 *ixg* folic acid/d during pregnancy and 200 mg Ca/d as placebo during the first 6 mo postpartum, with no supplementation for the offspring; MMN group: infants of women assigned to multiple micronutrient capsules containing 18 vitamins and minerals (including 20 mg Fe/d) during pregnancy and the first 6 mo postpartum, with no supplementation for the offspring; LNS group: infants of women assigned to 20 g LNS/d with the same micronutrients as the MMN group during pregnancy and the first 6 mo postpartum, and with the offspring assigned to 20 g LNS/d (for infants) containing 6 mg Fe/d from 6 to 18 mo of age. The LNS products contained calcium, phosphorous, potassium, and magnesium as well as macronutrients. All supplements were intended for daily consumption. IFA, iron and folic acid; LNS, lipid-based nutrient supplement; LPRR, longitudinal prevalence rate ratio; MMN, multiple micronutrient supplement.

2 Longitudinal prevalence is the percentage of infant-days of follow-up in which infants had morbidity events. LPRR is the ratio of the longitudinal prevalence of caregiver-reported morbidity symptoms in the treatment group to the longitudinal prevalence of caregiver-reported morbidity symptoms in the comparison group. Longitudinal prevalence and LPRR (95% CI) were generated using log-linear least-squares regression (SAS PROC GLIMMIX).

3 Noninferiority is concluded.

**Table 4:** Incidence of caregiver-reported nonscheduled visits to a “treatment point” due to morbidity events during 0-6 mo of age among infants born to pregnant women enrolled in the iLiNS-DYAD-Ghana micronutrient supplementation trial, by group according to supplements women were assigned to when enrolled^1^

	Incidence (per 100 infant-days), by group^2^			IRR of pairwise groups (95% CI)^2^		
	IFA (63,119 infant-days)	MMN (65,054 infant-days)	LNS (62,330 infant-days)	MMN vs IFA	LNS vs IFA	LNS vs MMN
All nonscheduled visits	1.03	0.99	0.95	0.97 (0.81,1.16)^3^	0.92 (0.76,1.10)^3^	0.96 (0.79,1.15)^3^
Due to acute respiratory infection	0.32	0.31	0.29	0.98 (0.74,1.25)	0.91 (0.70,1.19)^3^	0.95 (0.72,1.23)
Due to gastroenteritis	0.17	0.20	0.18	1.21 (0.83,1.76)	1.09 (0.74,1.60)	0.90 (0.62,1.30)
Due to fever/suspected malaria	0.21	0.18	0.21	0.83 (0.57,1.21)^3^	1.01 (0.70,1.46)	1.20 (0.83,1.75)
Due to “other illness”	0.21	0.19	0.15	0.91 (0.59,1.38)	0.74 (0.48,1.14)^3^	0.82 (0.53,1.27)
Due to poor appetite	0.03	0.01	0.02	0.41 (0.15,1.10)^3^	0.59 (0.24,1.42)	1.44 (0.48, 4.33)

1 *n* = 397, 403, and 397 for the IFA, MMN, and LNS groups, respectively. IFA group: infants of women assigned to 60 mg Fe/d and 400 ig folic acid/d during pregnancy and 200 mg Ca/d as placebo during the first 6 mo postpartum, with no supplementation for the offspring; MMN group: infants of women assigned to multiple micronutrient capsules containing 18 vitamins and minerals (including 20 mg Fe/d) during pregnancy and the first 6 mo postpartum, with no supplementation for the offspring; LNS group: infants of women assigned to 20 g LNS/d with the same micronutrients as the MMN group during pregnancy and the first 6 mo postpartum, and with the offspring assigned to 20 g LNS/d (for infants) containing 6 mg Fe/d from 6 to 18 mo of age. The LNS products contained calcium, phosphorous, potassium, and magnesium as well as macronutrients. Treatment centers are defined to include hospitals, clinics, health posts, pharmacies, private physicians, nurses, midwives, drug stores not operated by pharmacists, and traditional healers, typically used by residents of the area. There is no row for diarrhea based on mothers’ definition because visits to treatment points due to caregivers’ diagnosis of diarrhea overlapped with those due to gastroenteritis. We elected to show visits due to gastroenteritis only, to avoid double-reporting. IFA, iron and folic acid; IRR, incidence rate ratio; LNS, lipid-based nutrient supplement; MMN, multiple micronutrient supplement.

2 Incidence (per 100 infant-days) was calculated by dividing the number of treatment point visits by the total number of follow-up days, and multiplying the results by 100. IRR is the ratio of the incidence in the treatment group to the incidence in the comparison group. Incidence and IRR (95% CI) were generated using negative binomial regression models (SAS PROC GLIMMIX) and adjusting for the number of follow-up days for each child.

3 Noninferiority is concluded.

From 6 to 18 mo of age, the infants in the IFA and MMN groups combined contributed 240,097 infant-days of follow-up, whereas those in the LNS group contributed 118,698 infant- days of follow-up. During this period, the mean incidence and longitudinal prevalence of most of the morbidity events in the LNS group were noninferior to the corresponding mean incidence and longitudinal prevalence in the IFA + MMN groups (non-LNS group) combined (Table 5). Results for the incidence of “other illness” and poor appetite, and the longitudinal prevalence of these 2 symptom categories as well as fever/suspected malaria and diarrhea (caregiver report) were inconclusive, with the upper bounds of the 95% CIs of the IRRs exceeding the 1.2 noninferiority threshold by 0.06-0.09, and the upper bounds of the 95% CIs of the LPRRs exceeding the noninferiority threshold by 0.06-0.18. As shown in Table 6, the LNS group was noninferior to the non-LNS group with regard to the overall nonscheduled visits to a treatment point, or such visits resulting from ARI, gastroenteritis, or poor appetite. Noninferiority of the LNS group compared with the non-LNS group could not be established with respect to the incidence of visits to a treatment point because of fever/suspected malaria and “other reasons.”

**TABLE 5:** Incidence and longitudinal prevalence of caregiver-reported morbidity events during 6-18 mo of age among infants born to the pregnant women enrolled in the iLiNS-DYAD-Ghana micronutrient supplementation trial, by group according to supplements women were assigned to when enrolled^1^

	Incidence (per 100 infant-days)^2^			Longitudinal prevalence^3^		
	IFA + MMN (240,097 infant-days)	LNS (118,698 infant-days)	IRR (95% CI)	IFA + MMN (240,097 infant-days)	LNS (118,698 infant-days)	LPRR (95% CI)
All morbidity events combined	4.25	4.32	1.02 (0.96,1.08)^4^	28.23	29.29	1.04 (0.96,1.12)^4^
Acute respiratory infection	3.43	3.50	1.02 (0.94,1.11)^4^	19.90	20.54	1.03 (0.94,1.13)^4^
Diarrhea (caregiver-defined)	0.79	0.83	1.05 (0.93,1.19)^4^	3.12	3.34	1.07 (0.91, 1.26)
Gastroenteritis	0.88	0.89	1.02 (0.90,1.15)^4^	2.64	2.58	0.98 (0.84,1.14)^4^
Fever/suspected malaria	0.81	0.87	1.07 (0.95,1.19)^4^	1.77	2.00	1.13 (0.98,1.31)
Other illness	0.20	0.19	0.94 (0.71,1.26)	0.78	0.64	0.83 (0.55,1.25)
Poor appetite	1.00	1.18	1.16 (1.05,1.29)	7.39	8.75	1.18 (1.02,1.38)

1 *n* = 797 and 391 for the IFA + MMN and LNS groups, respectively. IFA + MMN, IFA group and MMN group combined; IFA group: infants of women assigned to 60 mg Fe/d and 400 ig folic acid/d during pregnancy and 200 mg Ca/d as placebo during the first 6 mo postpartum, with no supplementation for the offspring; MMN group: infants of women assigned to multiple micronutrient capsules containing 18 vitamins and minerals (including 20 mg Fe/d) during pregnancy and the first 6 mo postpartum, with no supplementation for the offspring; LNS group: infants of women assigned to 20 g LNS/d with the same micronutrients as the MMN group during pregnancy and the first 6 mo postpartum, and with the offspring assigned to 20 g LNS/d (for infants) containing 6 mg Fe/d from 6 to 18 mo of age. The LNS products also contained calcium, phosphorous, potassium, and magnesium as well as macronutrients. All supplements were intended for daily consumption. IFA, iron and folic acid; IRR, incidence rate ratio; LNS, lipid-based nutrient supplement; LPRR, longitudinal prevalence rate ratio; MMN, multiple micronutrient supplement.

2 Incidence (per 100 infant-days) was calculated by dividing the number of morbidity episodes by the total number of days at risk and multiplying the results by 100. IRR is the ratio of the incidence in the treatment group to the incidence in the comparison group. Incidence and IRR (95% CI) were generated using negative binomial regression models (SAS PROC GLIMMIX) and adjusting for the number of follow-up days for each child.

3 Longitudinal prevalence is the percentage of infant-days of follow-up in which infants had morbidity events. LPRR is the ratio of the longitudinal prevalence of caregiver-reported morbidity symptoms in the treatment group to the longitudinal prevalence of caregiver-reported morbidity symptoms in the comparison group. Longitudinal prevalence and LPRR (95% CI) were generated using log-linear least-squares regression (SAS PROC GLIMMIX).

4 Noninferiority is concluded.

**TABLE 6:** Incidence of caregiver-reported nonscheduled visits to a “treatment point” due to all morbidity events combined and to specific morbidity events during 6-18 mo of age among infants born to the pregnant women enrolled in the iLiNS-DYAD-Ghana micronutrient supplementation trial, by group according to supplements women were assigned to when enrolled^1^

	Incidence (per 100 infant-days)^2^		
	IFA + MMN (240,097 infant-days)	LNS (118,698 infant-days)	IRR (95%CI)
All nonscheduled visits	1.68	1.71	1.03 (0.94,1.12)^3^
Due to acute respiratory infection	0.40	0.39	0.98 (0.84,1.14)^3^
Due to gastroenteritis	0.33	0.31	0.93 (0.78,1.10)^3^
Due to fever/suspected malaria	0.55	0.62	1.12 (0.99,1.27)
Due to poor appetite	0.05	0.04	0.75 (0.50,1.13)^3^
Other reasons	0.16	0.16	1.00 (0.78,1.28)

1 *n* = 797 and 391 for the IFA + MMN and LNS groups, respectively. IFA + MMN, IFA group and MMN group combined; IFA group: infants of women assigned to 60 mg Fe/d and 400 µg folic acid/d during pregnancy and 200 mg Ca/d as placebo during the first 6 mo postpartum, with no supplementation for the offspring; MMN group: infants of women assigned to multiple micronutrient capsules containing 18 vitamins and minerals (including 20 mg Fe/d) during pregnancy and the first 6 mo postpartum, with no supplementation for the offspring; LNS group: infants of women assigned to 20 g LNS/d with the same micronutrients as the MMN group during pregnancy and the first 6 mo postpartum, and with the offspring assigned to 20 g LNS/d (for infants) containing 6 mg Fe/d from 6 to 18 mo of age. The LNS products also contained calcium, phosphorous, potassium, and magnesium as well as macronutrients. All supplements were intended for daily consumption. Treatment points are defined to include hospitals, clinics, health posts, private physicians, pharmacists, nurses, midwives, drug stores not operated by pharmacists, and traditional healers, typically used by residents of the area. There is no row for diarrhea based on mothers’ definition because visits to treatment points due to caregivers’ diagnosis of diarrhea overlapped with those due to gastroenteritis. We elected to show visits due to gastroenteritis only, to avoid double-reporting. IFA, iron and folic acid; IRR, incidence rate ratio; LNS, lipid-based nutrient supplement; MMN, multiple micronutrient supplement.

2 Incidence (per 100 infant-days) was calculated by dividing the number of morbidity episodes by the total number of follow-up days, and multiplying the results by 100. IRR is the ratio of the incidence in the treatment group to the incidence in the comparison group. Incidence and IRR (95% CI) were generated using negative binomial regression models (SAS PROC GLIMMIX) and adjusting for the number of follow-up days for each child.

3 Noninferiority is concluded.

## Discussion

In this semiurban setting in Ghana, infant morbidity during the first 6 mo of life was not increased by the provision of any of the maternal supplements (IFA, MMN, or SQ-LNS), compared with infants whose mothers received either of the other 2 supplements. Similarly, child incidence and longitudinal prevalence of all morbidity events (combined) between 6 and 18 mo of age were not increased by the provision of SQ- LNSs to mothers and then to their offspring from 6 to 18 mo, compared with children who did not receive SQ-LNSs and whose mothers received IFA or MMNs, nor was the incidence of ARI, diarrhea, gastroenteritis, or fever/suspected malaria. We could not conclude noninferiority with regard to the prevalence of diarrhea and fever/suspected malaria, nor the incidence or prevalence of poor appetite, when comparing children provided with SQ-LNSs with those not provided with any supplements, but those results were close to the noninferiority margin threshold of 1.2.

Our results that SQ-LNS consumption did not increase overall child morbidity agree with those from the iLiNS-DOSE trial in Malawi (19), in which the incidence of all caregiver- reported diseases combined was not greater in children assigned to 10, 20, or 40 g LNS/d during 6-18 mo of age, than in the control group which received no supplementation. Similar results were found in the iLiNS-DOSE trial (19) for longitudinal prevalence of all childhood morbidity symptoms combined (19), except for the group receiving 20 g LNS/d, in which noninferiority could not be concluded compared with control. In the iLiNS-DOSE trial, the provision of LNSs to infants did not decrease breastmilk intakes at 9-10 mo of age (48), and in Ghana, Burkina Faso, and Malawi, LNS consumption had no adverse effects on reported infant and young child feeding practices (continued breastfeeding or the frequency of breastfeeding) at 18 mo of age (49). The lack of adverse effects of SQ-LNS consumption on overall morbidity in our sample is consistent with the finding that SQ-LNSs do not reduce the intakes of breastmilk and its bioactive components that offer protection against infectious diseases (50, 51).

With regard to caregiver-reported ARI, diarrhea, gastroenteritis, and fever/suspected malaria, our results are also generally comparable with those of other LNS supplementation trials. For ARI, our findings of noninferiority of the LNS group in incidence and prevalence during 0-6 mo of age and 6-18 mo of age agree with those from Ghana (12), Malawi (15, 19), Haiti (52), Bangladesh (14, 20), Zimbabwe (53), and South Africa (18), which showed no group differences or demonstrated noninferiority of the LNS group compared with controls in the incidence or prevalence of cough (12, 15, 19, 20,52), nasal discharge (12,19, 20), rapid or difficult breathing (20, 53), or a combination of various acute respiratory symptoms regardless of fever (12, 14, 15, 19, 52). In the South Africa study (18), the consumption of 20 g/d regular LNSs or LNSs containing additional n-3 and n-6 fatty acids, lysine, and phytase from 6 to 12 mo of age reduced the prevalence of cough and difficulty breathing, compared with no supplementation, but only the regular LNSs reduced the prevalence of nasal discharge and the incidence of difficulty breathing. The n-3 fatty content of the LNSs may be related to their beneficial effects on ARIs in the South Africa study (54).

For diarrhea, the pairwise comparisons of incidence and prevalence from birth to 6 mo of age suggest that the 3 groups were indistinguishable from each other with respect to those outcomes during that period. This is similar to results from Bangladesh (20), where diarrheal morbidity from birth to 6 mo of age did not differ between infants whose mothers received different nutrient supplements during pregnancy and postpartum, and the infants themselves received no supplementation. During 6-18 mo of age, our results for the LNS compared with non-LNS groups add to the mixed findings across studies regarding the association between LNS consumption and diarrheal morbidity in children. Whereas similar findings of noninferiority of the LNS group in the incidence of diarrhea were reported in Bangladesh (14) and Burkina Faso (13), contrasting findings were reported in Malawi (15), where noninferiority to no supplementation could not be concluded, and South Africa (18), where 2 types of LNS products both increased the incidence of diarrhea. With regard to diarrheal prevalence, the upper limit of the 95% CI of the LPRR when comparing the LNS with the non-LNS group in our study (0.9, 1.3) was very close to 1.2 (Table 5), so our results are in general agreement with those of other studies showing no group differences (12, 13, 52, 53, 55) or a decrease (17), but not with others in which LNS consumption increased the prevalence of diarrhea (18, 20). The results for gastroenteritis follow the same pattern as those for diarrhea: first, maternal supplementation did not seem to affect the incidence or prevalence from birth to 6 mo of age when the infants did not consume any supplements directly; and second, the results for 6-18 mo of age (noninferiority of LNSs in the incidence and prevalence of gastroenteritis) agree with some previous findings (19) and contrast with others (18).

There are several potential explanations for the differences in results across studies regarding LNS consumption and diarrheal morbidity or gastroenteritis in children. These include variability in the definition of diarrheal episodes across studies (56), possible over-reporting when group allocations could not be blinded (18), and contextual factors linked to diarrheal morbidity, such as poor household water quality (57) and environmental sanitation (58, 59), cultural norms, and poverty Other possible factors affecting the risk of diarrhea or gastroenteritis among children consuming LNSs, which may vary by setting, include the effects of unabsorbed iron on the presence of pathogenic bacteria in the gut (60, 61) or the irritant effect of iron on gut motility (62, 63).

Regarding fever/suspected malaria, our results are generally consistent with previous reports from Ghana (12), Haiti (52), Bangladesh (20), Malawi (15, 19), and South Africa (18). In those studies, the LNS groups (including the 2 LNS formulations used in South Africa) did not differ (12, 20, 52), or were noninferior (15, 19), or had a lower (18) prevalence of fever/suspected malaria compared with controls, or the LNS group did not differ (18) or was noninferior (15, 19) in incidence of fever/suspected malaria compared with controls. Although there have been concerns about providing iron via LNSs in malaria-endemic regions such as Ghana, current WHO recommendations (64) indicate that iron supplementation may be implemented in malaria-endemic regions provided that the supplementation is done along with public health measures to prevent, diagnose, and treat malaria. At the time of our study, various malaria-control interventions (e.g., distribution of insecticide-treated bed-nets, vector control) were routinely implemented in Ghana (65, 66). Furthermore, the relatively small daily dose of iron (6.0 mg/d) provided in the 20 g SQ- LNS/d, which was given to children in two 10-g portions per day mixed with home-prepared complementary food, might reduce the likelihood of adverse effects, compared with the provision of a larger bolus of iron (67).

This study had several strengths and weaknesses. Strengths include a fully randomized design with active control groups, and intense weekly morbidity data collection along with caregivers’ use of a calendar grid to record infants’ morbidity and adherence to treatment. The study was originally designed as a superiority trial (for the primary outcomes) that maintained blinding to the group assignments to the maximum extent possible, and the noninferiority margin used in the present analysis was prespecified in our SAP. Such conditions are associated with less bias in results (68). The consumption of unintended supplements by some women in the IFA and MMN groups may potentially be a weakness of the study, but as previously reported (25), the percentage of follow- up days during which the unintended exposure occurred was relatively small (13%), and no women in the LNS group consumed any other supplement apart from the intended SQ- LNSs. We did not perform diagnostic tests for malaria at the home visits, and therefore we do not know the extent to which the caregivers’ reports or recalls of children’s fever or suspected malaria reflected the incidence or prevalence of “true malaria” in our sample. It is possible that there was bias in caregiver reports of the morbidity symptoms, despite using the morbidity calendar in most cases. This is particularly important because blinding of mothers between the LNS and non-LNS groups was not possible. In the South Africa study (18), the authors speculated that the inability to blind mothers to being in the 2 LNS groups as opposed to the no-supplement group may have contributed to over-reporting of diarrheal incidence in the LNS groups. A similar over-reporting of morbidity in the LNS group due to absence of blinding may have occurred in our study. We analyzed several prespecified secondary outcomes simultaneously, and it is possible that some of our findings may be due to chance, because of multiple testing (69). These prespecified secondary outcomes were, however, measured together at the same time (70) and highly correlated (71). Under such circumstances, correcting for multiplicity was considered unnecessary (71). The lower mean housing index for the children who completed the study than for the dropouts (suggesting lower background socioeconomic status of the former) could limit the generalizability of our results, although these groups did not differ in the other 2 socioeconomic variables, namely assets index and Household Food Insecurity Access Scale score.

In conclusion, our data support the hypothesis that in this semiurban setting in Ghana, the provision of SQ-LNSs to women from early pregnancy to 6 mo postpartum, and to their infants from 6 to 18 mo of age, does not increase the infants’ reported overall morbidity from birth to 6 mo of age, or from 6 to 18 mo of age. Further research is needed to understand contextual differences in the effects of nutritional supplements on diarrhea and malaria between children in different study settings.

## References

[1] Bailey RL, West KP, Jr Black RE The epidemiology of global micronutrient deficiencies. Ann Nutr Metab 2015;66(Suppl 2):22–33. 10.1159/000371618 26045325

[2] Fall CH, Fisher DJ, Osmond C, Margetts BM. Maternal Micronutrient Supplementation Study Group Multiple micronutrient supplementation during pregnancy in low-income countries: a metaanalysis of effects on birth size and length of gestation. Food Nutr Bull 2009;30:S533–6. 20120795 10.1177/15648265090304S408 PMC3541502

[3] Haider BA, Bhutta ZA Multiple-micronutrient supplementation for women during pregnancy. Cochrane Database Syst Rev 2012;11:CD004905. 23152228 10.1002/14651858.CD004905.pub3

[4] Ramakrishnan U, Grant FK, Goldenberg T, Bui V, Imdad A, Bhutta ZA Effect of multiple micronutrient supplementation on pregnancy and infant outcomes: a systematic review. Paediatr Perinat Epidemiol 2012;26(Suppl 1):153–67. 22742608 10.1111/j.1365-3016.2012.01276.x

[5] Iannotti LL, Tielsch JM, Black MM, Black RE Iron supplementation in early childhood: health benefits and risks. Am J Clin Nutr 2006;84:1261–76. 10.1093/ajcn/84.6.1261 PMC3311916 17158406

[6] WHO Resolution WHA 65.6. Comprehensive implementation plan on maternal, infant and young child nutrition. In: Sixty-fifth World Health Assembly, Geneva, 21-26 May 2012. Resolutions and decisions, annexes [Internet] Geneva (Switzerland): World Health Organization; 2012 [cited 2019 Aug 10]. Available from: http://www.who.int/nutrition/topics/WHA65.6_resolution_en.pdf?ua=1.

[7] WHO Global targets 2025. To improve maternal, infant and young child nutrition [Internet]. Geneva (Switzerland): World Health Organization; 2012 [cited 2019 Aug 10]. Available from: http://www.who.int/nutrition/topics/English_Poster_B_Global_Target_2025.pdf?ua=1.

[8] Arimond M, Zeilani M, Jungjohann S, Brown KH, Ashorn P, Allen LH, Dewey KG Considerations in developing lipid-based nutrient supplements for prevention of undernutrition: experience from the International Lipid-Based Nutrient Supplements (iLiNS) Project. Matern Child Nutr 2015;11(Suppl 4):31–61. 10.1111/mcn.12049 PMC6860325 23647784

[9] Das JK, Hoodbhoy Z, Salam RA, Bhutta AZ, Valenzuela-Rubio NG, Weise Prinzo Z, Bhutta ZA Lipid-based nutrient supplements for maternal, birth, and infant developmental outcomes. Cochrane Database Syst Rev 2018;8:CD012610. 30168868 10.1002/14651858.CD012610.pub2 PMC6513224

[10] Das JK, Salam RA, Hadi YB, Sadiq Sheikh S, Bhutta AZ, Weise Prinzo Z, Bhutta ZA Preventive lipid-based nutrient supplements given with complementary foods to infants and young children 6 to 23 months of age for health, nutrition, and developmental outcomes. Cochrane Database Syst Rev 2019;5:CD012611. 31046132 10.1002/14651858.CD012611.pub3 PMC6497129

[11] Sazawal S, Black RE, Ramsan M, Chwaya HM, Stoltzfus RJ, Dutta A, Dhingra U, Kabole I, Deb S, Othman MK, et al Effects of routine prophylactic supplementation with iron and folic acid on admission to hospital and mortality in preschool children in a high malaria transmission setting: community-based, randomised, placebo- controlled trial. Lancet 2006;367:133–43. 16413877 10.1016/S0140-6736(06)67962-2

[12] Adu-Afarwuah S, Lartey A, Brown KH, Zlotkin S, Briend A, Dewey KG Randomized comparison of 3 types of micronutrient supplements for home fortification of complementary foods in Ghana: effects on growth and motor development. Am J Clin Nutr 2007;86:412–20. 17684213 10.1093/ajcn/86.2.412

[13] Hess SY, Abbeddou S, Jimenez EY, Some JW, Vosti SA, Ouedraogo ZP, Guissou RM, Ouedraogo JB, Brown KH Small-quantity lipid-based nutrient supplements, regardless of their zinc content, increase growth and reduce the prevalence of stunting and wasting in young Burkinabe children: a cluster-randomized trial. PLoS One 2015;10:e0122242. 25816354 10.1371/journal.pone.0122242 PMC4376671

[14] Christian P, Shaikh S, Shamim AA, Mehra S, Wu L, Mitra M, Ali H, Merrill RD, Choudhury N, Parveen M, et al Effect of fortified complementary food supplementation on child growth in rural Bangladesh: a cluster-randomized trial. Int J Epidemiol 2015;44:1862–76. 26275453 10.1093/ije/dyv155 PMC4689999

[15] Mangani C, Ashorn P, Maleta K, Phuka J, Thakwalakwa C, Dewey K, Manary M, Puumalainen T, Cheung YB Lipid-based nutrient supplements do not affect the risk of malaria or respiratory morbidity in 6- to 18-month-old Malawian children in a randomized controlled trial. J Nutr 2014;144:1835–2. 25332483 10.3945/jn.114.196139

[16] Huybregts L, Houngbe F, Salpeteur C, Brown R, Roberfroid D, Ait- Aissa M, Kolsteren P The effect of adding ready-to-use supplementary food to a general food distribution on child nutritional status and morbidity: a cluster-randomized controlled trial. PLoS Med 2012;9:e1001313. 23028263 10.1371/journal.pmed.1001313 PMC3445445

[17] Luby SP, Rahman M, Arnold BF, Unicomb L, Ashraf S, Winch PJ, Stewart CP, Begum F, Hussain F, Benjamin-Chung J, et al Effects of water quality, sanitation, handwashing, and nutritional interventions on diarrhoea and child growth in rural Bangladesh: a cluster randomised controlled trial. Lancet Glob Health 2018;6:e302–e15. 29396217 10.1016/S2214-109X(17)30490-4 PMC5809718

[18] Smuts CM, Matsungo TM, Malan L, Kruger HS, Rothman M, Kvalsvig JD, Covic N, Joosten K, Osendarp SJM, Bruins MJ, et al Effect of small- quantity lipid-based nutrient supplements on growth, psychomotor development, iron status, and morbidity among 6- to 12-mo-old infants in South Africa: a randomized controlled trial. Am J Clin Nutr 2019;109(1):55–68. 30649163 10.1093/ajcn/nqy282 PMC6358035

[19] Bendabenda J, Alho L, Ashorn U, Cheung YB, Dewey KG, Vosti SA, Phuka J, Maleta K, Ashorn P The effect of providing lipid-based nutrient supplements on morbidity in rural Malawian infants and young children: a randomized controlled trial. Public Health Nutr 2016;19:1893–903. 26956611 10.1017/S1368980016000331 PMC10271160

[20] Ullah MB, Mridha MK, Arnold CD, Matias SL, Khan MSA, Siddiqui Z, Hossain M, Dewey KG Provision of pre- and postnatal nutritional supplements generally did not increase or decrease common childhood illnesses in Bangladesh: a cluster-randomized effectiveness trial. J Nutr 2019;149:1271–81. 31162588 10.1093/jn/nxz059

[21] GSS/GHS/ICF International Ghana Demographic and Health Survey [Internet]. Accra (Ghana) and Rockville (MD): Ghana Statistical Service (GSS), Ghana Health Service (GHS), and ICF International; [cited 2019 Aug 7]. Available from: https://dhsprogram.com/pubs/pdf/FR307/FR307.pdf.

[22] University of Ghana/GroundWork/University of Wisconsin-Madison/KEMRI-WellcomeTrust/UNICEF Ghana Micronutrient Survey 2017 [Internet]. Accra, Ghana: UNICEF-Accra; 2017 [cited 2019 May 21]. Available from: http://groundworkhealth.org/wp-content/uploads/2018/06/UoG-GroundWork_2017-GHANA-MICRONUTRIENT-SURVEY_Final_180607.pdf.

[23] Amugsi DA, Aborigo RA, Oduro AR, Asoala V, Awine T, Amenga-Etego L Socio-demographic and environmental determinants of infectious disease morbidity in children under 5 years in Ghana. Glob Health Action 2015;8:29349. 26455493 10.3402/gha.v8.29349 PMC4600709

[24] Adu-Afarwuah S, Lartey A, Okronipa H, Ashorn P, Zeilani M, Peerson JM, Arimond M, Vosti S, Dewey KG Lipid-based nutrient supplement increases the birth size of infants of primiparous women in Ghana. Am J Clin Nutr 2015;101:835–6. 25833980 10.3945/ajcn.114.091546

[25] Adu-Afarwuah S, Lartey A, Okronipa H, Ashorn P, Peerson JM, Arimond M, Ashorn U, Zeilani M, Vosti S, Dewey KG Small- quantity, lipid-based nutrient supplements provided to women during pregnancy and 6 mo postpartum and to their infants from 6 mo of age increase the mean attained length of 18-mo-old children in semi- urban Ghana: a randomized controlled trial. Am J Clin Nutr 2016;104: 797–808. 27534634 10.3945/ajcn.116.134692 PMC4997301

[26] Adu-Afarwuah S, Young RT, Lartey A, Okronipa H, Ashorn P, Ashorn U, Oaks BM, Arimond M, Dewey KG Maternal and infant supplementation with small-quantity lipid-based nutrient supplements increases infants’ iron status at 18 months of age in a semiurban setting in Ghana: a secondary outcome analysis of the iLiNS-DYAD randomized controlled trial. J Nutr 2019;149: 149–58. 30624674 10.1093/jn/nxy225 PMC6351141

[27] WHO Guideline: daily iron and folic acid supplementation in pregnant women [Internet]. Geneva (Switzerland): World Health Organization; 2012 [cited 2019 Aug 13]. Available from: http://apps.who.int/iris/bitstream/10665/77770/1/9789241501996_eng.pdf?ua=1. 23586119

[28] UNICEF/WHO/United Nations University Composition of a multi-micronutrient supplement to be used in pilot programmes among pregnant women in developing countries [Internet]. New York: UNICEF; 1999 [cited 2015 Jan 18]. Available from:http://apps.who.int/iris/bitstream/10665/75358/1/UNICEF-WHO-multi-micronutrients.pdf?ua=1..

[29] Zhou SJ, Gibson RA, Crowther CA, Makrides M Should we lower the dose of iron when treating anaemia in pregnancy? A randomized dose- response trial. Eur J Clin Nutr 2009;63:183–90. 17928802 10.1038/sj.ejcn.1602926

[30] Institute of Medicine Dietary Reference Intakes for vitamin A, vitamin K, arsenic, boron, chromium, copper, iodine, iron, manganese, molybdenum, nickel, silicon, vanadium and zinc. Washington (DC): National Academy Press; 2001. 25057538

[31] WHO/UNICEF Iron supplementation of young children in regions where malaria transmission is intense and infectious disease highly prevalent [Internet]. Geneva (Switzerland): World Health Organization; 2006 [cited 2019 Aug 14]. Available from: http://www.who.int/malaria/publications/atoz/who_statement_iron/en/.

[32] Arnold BF, Galiani S, Ram PK, Hubbard AE, Briceno B, Gertler PJ, Colford JM, Jr Optimal recall period for caregiver-reported illness in risk factor and intervention studies: a multicountry study. Am J Epidemiol 2013;177:361–70. 23364878 10.1093/aje/kws281

[33] Pan American Health Organization/WHO Guiding principles for complementary feeding of the breastfed child [Internet]. Washington (DC): Pan American Health Organization and World Health Organization; 2002 [cited 2019 May 21]. Available from: https://www.who.int/nutrition/publications/guiding_principles_compfeeding_breastfed.pdf.

[34] WHO The treatment of diarrhea. A manual for physicians and other senior health workers [Internet]. Geneva (Switzerland): World Health Organization; 2005 [cited 2018 May 1]. Available from: http://www.who.int/maternal_child_adolescent/documents/9241593180/en/.

[35] Cogswell ME, Oni GA, Stallings RY, Brown KH Sociodemographic and clinical factors affecting recognition of childhood diarrhea by mothers in Kwara State, Nigeria. Soc Sci Med 1991;33: 1209–16. 1767291 10.1016/0277-9536(91)90237-7

[36] Olson D, Lamb MM, Lopez MR, Paniagua-Avila MA, Zacarias A, Samayoa-Reyes G, Cordon-Rosales C, Asturias EJ Rapid active sampling surveys as a tool to evaluate factors associated with acute gastroenteritis and norovirus infection among children in rural Guatemala. Am J Trop Med Hyg 2017;97: 944–8. 28722580 10.4269/ajtmh.16-1003 PMC5590593

[37] Morris SS, Santos CA, Barreto ML, Cousens SN, Strina A, Santos LM, Assis AM Measuring the burden of common morbidities: sampling disease experience versus continuous surveillance. Am J Epidemiol 1998;147: 1087–92. 9620053 10.1093/oxfordjournals.aje.a009403

[38] Cohen J Statistical power analysis in the behavioral sciences. 2nd ed. Hillsdale (NJ): Lawrence Erlbaum Associates, Inc; 1988.

[39] Groenwold RH, Moons KG, Vandenbroucke JP Randomized trials with missing outcome data: how to analyze and what to report. CMAJ 2014;186: 1153–7. 24778353 10.1503/cmaj.131353 PMC4203602

[40] Klevor MK, Adu-Afarwuah S, Ashorn P, Arimond M, Dewey KG, Lartey A, Maleta K, Phiri N, Pyykko J, Zeilani M, et al A mixed method study exploring adherence to and acceptability of small quantity lipid-based nutrient supplements (SQ-LNS) among pregnant and lactating women in Ghana and Malawi. BMC Pregnancy Childbirth 2016;16:253. 27577112 10.1186/s12884-016-1039-0 PMC5004276

[41] Lemaire M, Islam QS, Shen H, Khan MA, Parveen M, Abedin F, Haseen F, Hyder Z, Cook RJ, Zlotkin SH Iron-containing micronutrient powder provided to children with moderate-to- severe malnutrition increases hemoglobin concentrations but not the risk of infectious morbidity: a randomized, double-blind, placebo- controlled, noninferiority safety trial. Am J Clin Nutr 2011;94:585–93. 21715512 10.3945/ajcn.110.009316

[42] Lange S, Freitag G Choice of delta: requirements and reality - results of a systematic review. Biom J 2005;47: 12–27.; discussion 99-107. 16395993 10.1002/bimj.200410085

[43] European Medicines Agency Guideline on the choice of the noninferiority margin [Internet]. London (UK): European Medicines Agency, Committee for Medicinal Products for Human Use; 2005 [cited 2019 Aug 4]. Available from: https://www.ema.europa.eu/en/documents/scientific-guideline/guideline-choice-non-inferiority-margin_en.pdf.

[44] FDA Non-inferiority clinical trials to establish effectiveness: guidance for industry [Internet]. Silver Spring (MD): Food and Drug Administration; 2016 [cited 2019 Aug 4]. Available from: https://www.fda.gov/media/78504/download.

[45] Piaggio G, Elbourne DR, Pocock SJ, Evans SJ, Altman DG; CONSORT Group. Reporting of noninferiority and equivalence randomized trials: extension of the CONSORT 2010 statement. JAMA 2012;308:2594–604. 23268518 10.1001/jama.2012.87802

[46] Macaya F, Ryan N, Salinas P, Pocock SJ Challenges in the design and interpretation of noninferiority trials: insights from recent stent trials. J Am Coll Cardiol 2017;70: 894–903. 28797360 10.1016/j.jacc.2017.06.039

[47] Nestel P; INACG Steering Committee. Adjusting hemoglobin values in program surveys [Internet]. Washington (DC): International Nutritional Anemia Consultative Group; 2002 [cited 2019 Jul 10]. Available from: http://pdf.usaid.gov/pdf_docs/PNACQ927.pdf.

[48] Kumwenda C, Dewey KG, Hemsworth J, Ashorn P, Maleta K, Haskell MJ Lipid-based nutrient supplements do not decrease breast milk intake of Malawian infants. Am J Clin Nutr 2014;99: 617–23. 24368436 10.3945/ajcn.113.076588

[49] Arimond M, Abbeddou S, Kumwenda C, Okronipa H, Hemsworth J, Jimenez EY, Ocansey E, Lartey A, Ashorn U, Adu-Afarwuah S, et al Impact of small quantity lipid-based nutrient supplements on infant and young child feeding practices at 18 months of age: results from four randomized controlled trials in Africa. Matern Child Nutr 2017;13:e12377. 10.1111/mcn.12377 PMC5516197 27910260

[50] Andreas NJ, Kampmann B, Mehring Le-Doare K Human breast milk: a review on its composition and bioactivity. Early Hum Dev 2015;91: 629–35. 26375355 10.1016/j.earlhumdev.2015.08.013

[51] Dixon DL The role of human milk immunomodulators in protecting against viral bronchiolitis and development of chronic wheezing illness. Children (Basel) 2015;2: 289–304. 27417364 10.3390/children2030289 PMC4928768

[52] Iannotti LL, Dulience SJ, Green J, Joseph S, Francois J, Antenor ML, Lesorogol C, Mounce J, Nickerson NM Linear growth increased in young children in an urban slum of Haiti: a randomized controlled trial of a lipid-based nutrient supplement. Am J Clin Nutr 2014;99:198–208. 24225356 10.3945/ajcn.113.063883 PMC3862455

[53] Humphrey JH, Mbuya MNN, Ntozini R, Moulton LH, Stoltzfus RJ, Tavengwa NV, Mutasa K, Majo F, Mutasa B, Mangwadu G, et al Independent and combined effects of improved water, sanitation, and hygiene, and improved complementary feeding, on child stunting and anaemia in rural Zimbabwe: a cluster-randomised trial. Lancet Glob Health 2019;7:e132–e47. 30554749 10.1016/S2214-109X(18)30374-7 PMC6293965

[54] Hageman JH, Hooyenga P, Diersen-Schade DA, Scalabrin DM, Wichers HJ, Birch EE The impact of dietary long-chain polyunsaturated fatty acids on respiratory illness in infants and children. Curr Allergy Asthma Rep 2012;12: 564–73. 23001718 10.1007/s11882-012-0304-1 PMC3492691

[55] Null C, Stewart CP, Pickering AJ, Dentz HN, Arnold BF, Arnold CD, Benjamin-Chung J, Clasen T, Dewey KG, Fernald LCH, et al Effects of water quality, sanitation, handwashing, and nutritional interventions on diarrhoea and child growth in rural Kenya: a cluster-randomised controlled trial. Lancet Glob Health 2018;6: e316–e29. 29396219 10.1016/S2214-109X(18)30005-6 PMC5809717

[56] Baqui AH, Black RE, Yunus M, Hoque AR, Chowdhury HR, Sack RB Methodological issues in diarrhoeal diseases epidemiology: definition of diarrhoeal episodes. Int J Epidemiol 1991;20: 1057–63. 1800404 10.1093/ije/20.4.1057

[57] Trevett AF, Carter RC, Tyrrel SF The importance of domestic water quality management in the context of faecal-oral disease transmission. J Water Health 2005;3: 259–70. 16209030 10.2166/wh.2005.037

[58] Baker KK, Senesac R, Sewell D, Sen Gupta A, Cumming O, Mumma J Fecal fingerprints of enteric pathogen contamination in public environments of Kisumu, Kenya, associated with human sanitation conditions and domestic animals. Environ Sci Technol 2018;52:10263–74. 30106283 10.1021/acs.est.8b01528 PMC6557411

[59] Pickering AJ, Ercumen A, Arnold BF, Kwong LH, Parvez SM, Alam M, Sen D, Islam S, Kullmann C, Chase C, et al Fecal indicator bacteria along multiple environmental transmission pathways (water, hands, food, soil, flies) and subsequent child diarrhea in rural Bangladesh. Environ Sci Technol 2018;52:7928–36. 29902374 10.1021/acs.est.8b00928 PMC7705120

[60] Jaeggi T, Kortman GA, Moretti D, Chassard C, Holding P, Dostal A, Boekhorst J, Timmerman HM, Swinkels DW, Tjalsma H, et al Iron fortification adversely affects the gut microbiome, increases pathogen abundance and induces intestinal inflammation in Kenyan infants. Gut 2015;64: 731–42. 25143342 10.1136/gutjnl-2014-307720

[61] Soofi S, Cousens S, Iqbal SP, Akhund T, Khan J, Ahmed I, Zaidi AK, Bhutta ZA Effect of provision of daily zinc and iron with several micronutrients on growth and morbidity among young children in Pakistan: a cluster-randomised trial. Lancet 2013;382: 29–40. 23602230 10.1016/S0140-6736(13)60437-7

[62] Gera T, Sachdev HP Effect of iron supplementation on incidence of infectious illness in children: systematic review. BMJ 2002;325:1142. 12433763 10.1136/bmj.325.7373.1142 PMC133452

[63] Zaim M, Piselli L, Fioravanti P, Kanony-Truc C Efficacy and tolerability of a prolonged release ferrous sulphate formulation in iron deficiency anaemia: a non-inferiority controlled trial. Eur J Nutr 2012;51:221–9. 21643774 10.1007/s00394-011-0210-7

[64] WHO Guideline: daily iron supplementation in infants and children [Internet]. Geneva (Switzerland): World Health Organization; 2016 [cited 2019 Apr 11]. Available from: http://apps.who.int/iris/bitstream/10665/204712/1/9789241549523_eng.pdf?ua=1&ua=1. 27195348

[65] Awine T, Malm K, Bart-Plange C, Silal SP Towards malaria control and elimination in Ghana: challenges and decision making tools to guide planning. Glob Health Action 2017;10:1381471. 29035160 10.1080/16549716.2017.1381471 PMC5678345

[66] Government of Ghana President’s malarial initiative, Ghana: Malaria Operational Plan FY 2018 [Internet]. Accra (Ghana): Government of Ghana; 2018 [cited 2018 Dec 10]. Available from: https://www.pmi.gov/docs/default-source/default-document-library/malaria-operational-plans/fy-2018/fy-2018-ghana-malaria-operational-plan.pdf?sfvrsn=5.

[67] Dewey KG, Baldiviez LM Safety of universal provision of iron through home fortification of complementary foods in malaria-endemic areas. Adv Nutr 2012;3: 555–9. 22797992 10.3945/an.111.001131 PMC3649726

[68] Oczkowski SJ A clinician’s guide to the assessment and interpretation of noninferiority trials for novel therapies. Open Med 2014;8:e67–72. 25009686 PMC4085087

[69] Li G, Taljaard M, Van den Heuvel ER, Levine MA, Cook DJ, Wells GA, Devereaux PJ, Thabane L An introduction to multiplicity issues in clinical trials: the what, why, when and how. Int J Epidemiol 2017;46:746–55. 28025257 10.1093/ije/dyw320

[70] Craig P, Dieppe P, Macintyre S, Michie S, Nazareth I, Petticrew M Developing and evaluating complex interventions: new guidance [Internet]. Swindon (UK): Medical Research Council, UK; 2006 [cited 2019 Jan 5]. Available from: http://www.mrc.ac.uk/documents/pdf/complex-interventions-guidance/.

[71] Streiner DL Best (but oft-forgotten) practices: the multiple problems of multiplicity—whether and how to correct for many statistical tests. Am J Clin Nutr 2015;102:721–8. 26245806 10.3945/ajcn.115.113548

